# Air-ozonolysis activation of polyolefins versus use of laden finishing to form contact-active nonwoven materials

**DOI:** 10.1038/s41598-021-90218-2

**Published:** 2021-05-24

**Authors:** Stella Kiel, Miri Klein, Yulia Kroupitski, Uri M. Peiper, Shlomo Sela Saldinger, Elena Poverenov

**Affiliations:** 1 Department of Food Science, Agro-Nanotechnology and Advanced Materials Research Center, Agricultural Research Organization, The Volcani Center, 7505101 Rishon Lezion, Israel; 2 Department of Agricultural Engineering, Agricultural Research Organization, The Volcani Center, 7505101 Rishon Lezion, Israel

**Keywords:** Health care, Chemistry, Engineering, Materials science

## Abstract

Two synthetic approaches were explored for modification of the polyolefins polyethylene/polypropylene (PE/PP) to form contact-active nonwoven materials. In the first approach, polymer surfaces were activated by O_2_-free air-ozonolysis, and then the active agent (trimethoxysilyl) propyl-octadecyl-dimethyl-ammonium chloride (C18-TSA) was covalently bound. In the second approach, the active agent was directly conjugated to the commercial ‘finishing’ that was then applied to the polymer. The chemical, physical and microscopic properties of the modified polymers were comprehensively studied, and their active site density was quantified by fluorescein sodium salt-cetyltrimethylammonium chloride reaction. The antimicrobial activity of the prepared nonwovens against *Bacillus subtilis* (Gram-positive) and *Salmonella enterica* (Gram-negative), and their stability at various pHs and temperatures were examined. The two approaches conferred antimicrobial properties to the modified polymers and demonstrated stable linkage of C18-TSA. However, the performance of the nonwovens formed by the first approach was superior. The study suggests two feasible and safe pathways for the modification of polyolefins to form contact-active nonwoven materials that can be further applied in various fields, such as hygiene products, medical fabrics, sanitizing wipes, and more.

## Introduction

Polyethylene (PE) and polypropylene (PP) are the most widely used polyolefins for the production of nonwoven materials. The production of a new generation of active nonwovens that possess advanced beneficial properties is garnering a great deal of attention. These active nonwovens may have a vast range of applications in medicine, baby care and hygiene products, agriculture, sanitizing wipes, decorative and technical fabrics, and more^1^. Therefore, numerous researchers are focusing on the development of feasible methods for their synthesis. Deposition of nanostructured inorganic antibacterial agents, such as silver, titanium dioxide, copper, gold, zinc oxide, and gallium nanoparticles and carbon nanotubes, is a widely used approach to imparting antimicrobial capacity to polyolefin-based nonwovens^2–4^. Co-electrospinning technique, which applies an electric field on a polymer–active agent composite to produce active fibers, is also used^5,6^. Immobilization of macromolecules on the nonwoven's surface has also been reported. For instance, Lin et al.^7^ immobilized biocidal ε-polylysine and natamycin on polyethylene terephthalate using soft methacrylate adhesives, and Wang and Chen^8^ immobilized collagen/chitosan on acrylic acid-modified PP nonwovens.

Covalent linkage represents one of the most effective approaches for the formation of stable and reliable contact-active materials that, due to the absence of undesirable release and leaching, benefit from higher safety and economic viability^9–11^. PP and PE, however, lack the functional groups necessary for covalent linkage of active agents and must be preactivated. Plasma treatments for the activation of "inert" polymers, enabling their further modification, including the attachment of active agents, have been reported^12–15^. Tsou et al.^16^ suggested simultaneous plasma treatment and grafting by a process termed immersion–pad-pressing–drying–plasma to fabricate PP with antibacterial and hydrophilic surface properties. Plasma-aided activation can also be followed by grafting of the active moiety according to the designated purpose^17,18^. O_2_-sourced ozone treatment induces activation of polyolefins by adding oxygenated groups on their surface, allowing their further functionalization^19,20^. Despite the environmental friendliness of these activation methods, however, the application of plasma and O_2_–ozone treatments for mass production of modified polyolefin-based materials has some practical drawbacks. Available plasma reactors are only capable of producing a relatively small volume of plasma at low speed, whereas the use of O_2_ gas for ozone production in the ozone treatments raises safety concerns^20,21^. Thus, because polyolefin modification toward the formation of active materials is a process that still needs significant enhancement and rationalization, research in this field continues.

In this article, we present two synthetic approaches for the modification of polyethylene/polypropylene (PE/PP) with the quaternary ammonium salt (trimethoxysilyl)propyl-octadecyl-dimethyl-ammonium chloride (C18-TSA) for further formation of contact-active nonwovens. Quaternary ammonium salts are widely used as antimicrobial agents due to their robust and broad-spectrum activities^22,23^. In the first method, we utilize a safe and feasible air-ozonolysis method, which does not require O_2_ gas^24^, to activate the PE/PP surface toward covalent linkage of C18-TSA. In the second approach, the PE/PP surface is not activated; instead, C18-TSA is directly attached to a commercial finishing. Commercial finishings are consistently used in the nonwovens industry to enhance the resulting materials’ properties, such as softness, hydrophobicity, etc.^25,26^. To the best of our knowledge, this is the first time that a routine finishing treatment has been used for the preparation of antimicrobial nonwovens. The modified materials prepared by both methods were comprehensively characterized and their physical properties, active-site density, stability and antimicrobial activity were examined.

## Results

### Synthesis and characterization

**Figure 1:**
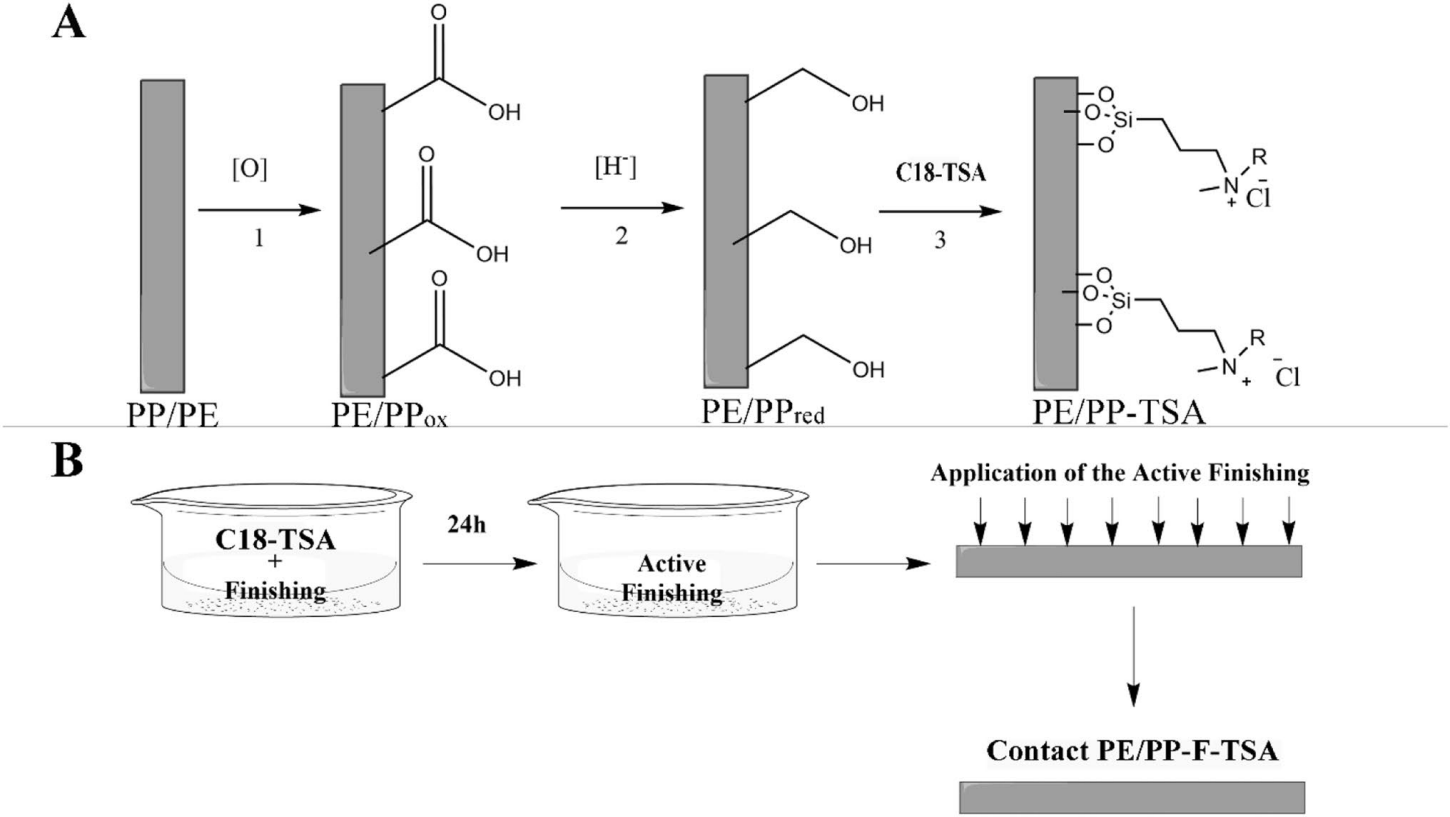
Schematic presentation of C18-TSA addition to PE/PP nonwovens. (**A**) Air-ozonolysis surface activation (1), reduction (2), and silylation (3) to form PE/PP-TSA. (**B**) Direct C18-TSA conjugation using commercial finishing to form PE/PP-F-TSA.

Two approaches were used to attach the quaternary ammonium salt C18-TSA to the polyolefins PE/PP. In the first approach, the air-ozonolysis method, which does not require O_2_ gas and therefore presents significant safety and practical advantages, was utilized for polymer surface oxidation to form PE/PP_ox_. The activated polymers were then reduced to PE/PP_red_ and subjected to silylation reaction with C18-TSA to form PE/PP-TSA. In the second approach, C18-TSA was attached directly to the commercial finishing and the resultant "active finishing" was applied to the polymer, forming PE/PP-F-TSA (Fig. 1).

**Figure 2:**
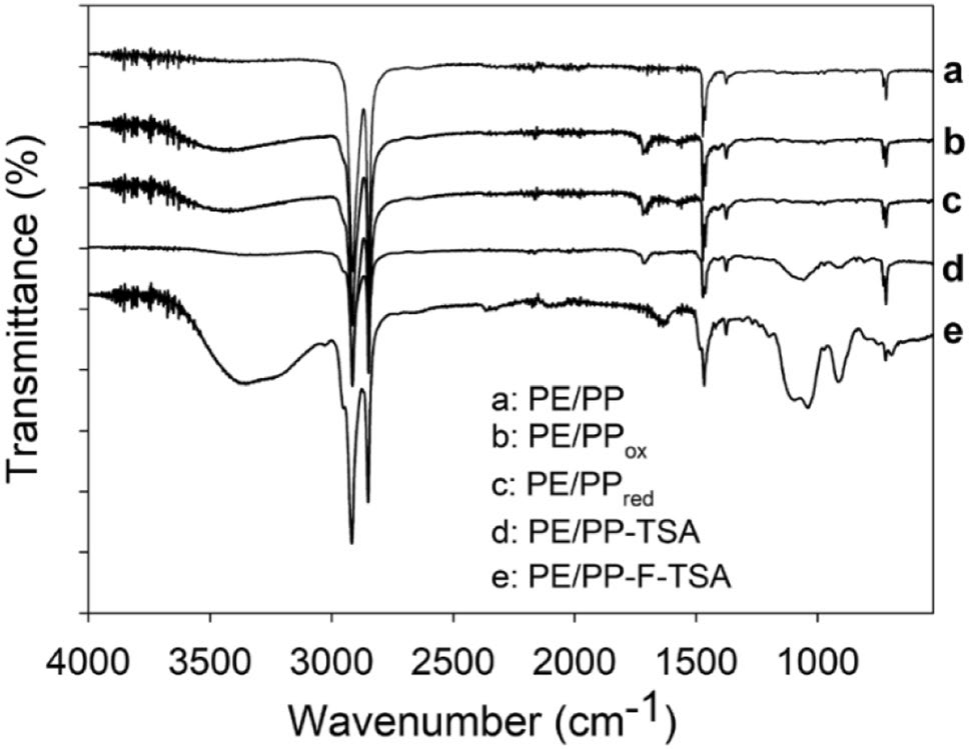
ATR-FTIR spectra of PE/PP, PE/PP_ox_, PE/PP_red_, PE/PP-TSA and PE/PP-F-TSA.

ATR-FTIR measurements confirmed C18-TSA grafting. In the first method, carboxylic group stretching bands at 1717 cm^−1^ appeared after air-ozonolysis (Fig. 2, curve b), pointing on successful activation of the polyolefins' surface. Upon C18-TSA conjugation, the PE/PP-TSA spectra presented a new peak associated with Si–O–C bond formation at 1060 cm^−1^, appearance of carboxylic peak in PE/PP-TSA indicates that not all the carboxylic groups were reduced and they exist in all stages (Fig. 2, curve d). The ATR-FTIR spectra of the PE/PP-F-TSA film treated with the active finishing also showed a new peak in the Si–O–C region (Fig. 2, curve e).

**Figure 3:**
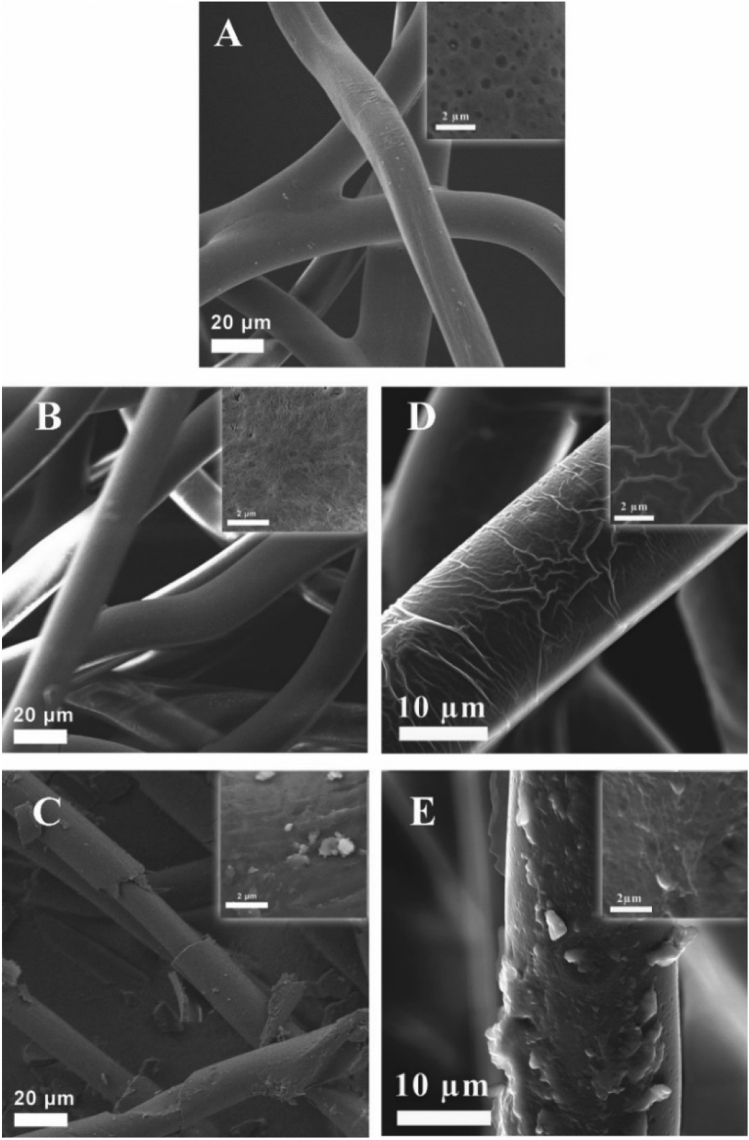
SEM images of PE/PP films treated by the two tested methods. (**A**) Pristine PE/PP, (**B**) PE/PP_ox_, (**C**) PE/PP-TSA, (**D**) PE/PP-F, and (**E**) PE/PP-F-TSA at the 30 k magnification; inset: 1.5 k magnification.

SEM analysis was performed to examine the surface morphology of the studied materials and to understand their distinctive properties (Fig. 3). The pristine PE/PP demonstrated a smooth, slightly porous surface. Activation by air-ozonolysis led to a more porous surface for PE/PP_ox_ due to the generation of carboxylic groups. Covalent linkage of C18-TSA resulted in covered fibers for the PE/PP-TSA film. These results correlated with those presented in our previous research^24^. The regular commercial finishing caused the appearance of a coating layer, while in the treatment with the active finishing, this layer was slightly less homogeneous, indicating adhesion of C18-TSA to the commercial finishing.

**Figure 4:**
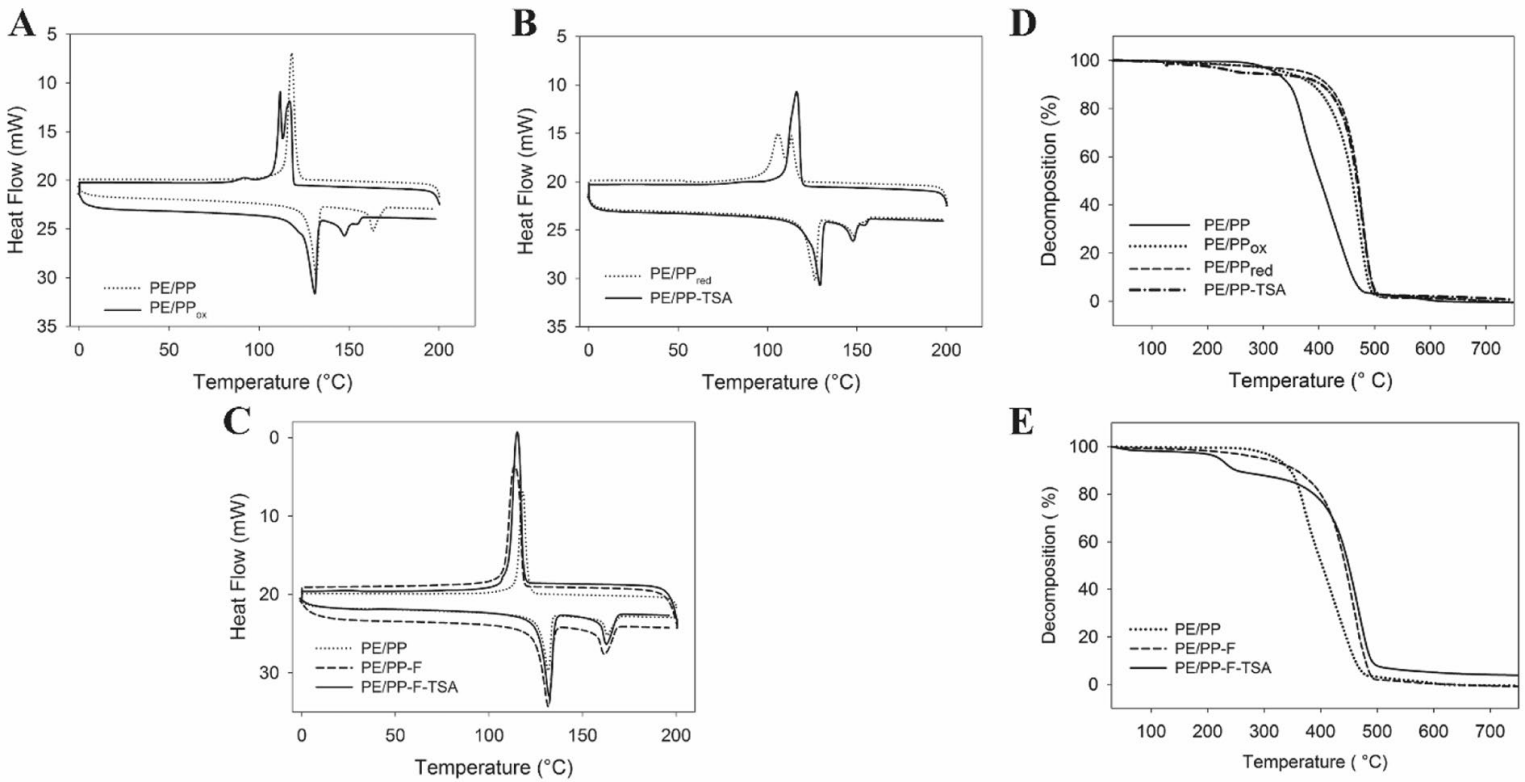
(Left) DSC thermograms of PE/PP modified using the air-ozonolysis activation method (**A**, **B**) and commercial finishing (**C**). (Right) TGA thermograms of PE/PP modified using the air-ozonolysis activation method (**D**) and the commercial finishing (**E**).

DSC thermograms of the pristine PE/PP exhibited exothermic peak onset and maximum temperature at 89 °C and 118 °C, respectively. The exothermic peak onset of PE/PP_ox_, PE/PP_red_ and PE/PP-TSA occurred between 101 and 117 °C, 73 and 113 °C, and 97 and 116 °C, respectively. Notably, DSC thermograms of the oxidized and reduced PE/PP demonstrated a splitting of the exothermic peaks, which might be associated with the oxidation and reduction processes (Fig. 4A,B). This peak splitting disappeared after conjugation of C18-TSA. Percent oxidation was calculated as the ratio of the areas under the exothermic peaks of the pristine PE/PP and oxidized PE/PP; percent reduction was calculated as the ratio of the areas under the exothermic peaks of the oxidized and reduced PE/PP (Table 1).

**Table 1:** DSC analysis of PE/PP, PE/PPox, PE/PPred, PE/PP-TSA, PE/PP-F, and PE/PP-F-TSA.

Materials	ΔH_1_ (J g^−1^)	ΔH_2_ (J g^−1^)	ΔH_3_ (J g^−1^)	Tm_1_ (°C)	Tm_2_ (°C)	Tc (°C)	Peak_1_ area (mJ)	Peak_2_ area (mJ)	Oxidation/reduction percentage (%)
PE/PP	− 130.8	80.6	27.9	102.6	143.7	118.2	− 719.2		
PE/PP_ox_	− 19.4	− 40.3		101.4	136.6		− 124.3	− 257.7	35.8
PE/PP_red_	− 34.1	− 21.6		101.3	133.3		− 197.6	− 125.4	48.7
PE/PP-TSA	− 132			103.4	134.8		− 765.5		
PE/PP-F	− 118.3	72.6	32.3	125.5	157.4	108.3			
PE/PP-F-TSA	− 130.8	84.0	33.0	126.5	159.1	110.3			

Materials	Average water contact angle (°)			Water-penetration time (s)			Water-penetration velocity (mL s^-1^)		
PE/PP	112.7 ± 4.3			2.55			0.255		
PE/PP_ox_	101.1 ± 4.1			17			3.4		
PE/PP_red_	108.5 ± 5.3			16			3.2		
PE/PP-TSA	120.8 ± 5.9			18			3.6		
PE/PP-F	94.6 ± 3.8			1.95			0.195		
PE/PP-F-TSA	111.5 ± 11.9			> 20			> 4		

Surface water contact angle and water-passage time for PE/PP, PE/PPox, PE/PPred, PE/PP-TSA, PE/PP-F, and PE/PP-F-TSA.

*ΔH*, *Tm*, *Tc* were measured by DSC, *Tm* refers to endothermic peak, *Tc* refers to exothermic peak.

The DSC thermogram of PE/PP-F-TSA showed two endothermic peaks. This implies that the melting process occurred in two steps, the first associated with PP melting, and the second with PE melting. Exothermic peaks of PE/PP-F and PE/PP-F-TSA in Fig. 4C evidenced partial crystallinity of the films.

In the TGA studies, the pristine PE/PP was thermally stable up to ∼280 °C; then decomposition started, reaching 96% at ∼482 °C. The PE/PP-TSA and PE/PP-F-TSA films demonstrated a similar pattern, showed a typical two-step degradation curve at 150–260 °C and 360–510 °C, due to the decomposition of quaternary ammonium and the pyrolysis of alkane chains, respectively (Fig. 4D,E). This decomposition pattern is typical of alkane-substituted quaternary ammonium salts ^27^.

The hydrophobicity of the prepared materials was evaluated by water contact angle measurements before and after conjugation of C18-TSA (Table 1). Due to the hydrophobic nature of TSA, its conjugation led to an increase in water contact angle for PE/PP-TSA compared to the pristine PE/PP, and for PE/PP-F-TSA compared to PE/PP-F (Table 1). The water-passage studies confirmed hydrophobicity enhancement upon conjugation of C18-TSA, with PE/PP-TSA and PE/PP-F-TSA demonstrating increased water-passage times (Table 1).

### Stability studies

TSA grafting stability was verified by TOC-L measurements of the released nitrogen content at 25 °C, 50 °C and 70 °C and pH 3, 7 and 11 (Table 2). Both modification methods led to stable materials that did not show any evidence of notable C18-TSA release at 25 or 50 °C, or at pH 3 or 7. Evidence of C18-TSA release was observed at 70 °C and/or pH 11 (Table 2). The PE/PP-TSA formed using air-ozonolysis activation was generally more stable than the PE/PP-F-TSA formed using commercial finishing (Table 2). This implies that the covalent conjugation of TSA to the activated PE/PP surfaces led to stronger binding.

**Table 2:** The release of N-content from PE/PP-TSA and PE/PP-F-TSA studied by TOC-L analyzer at various temperatures and pH conditions.

PE/PP-TSA		PE/PP-F-TSA	
Temperature (°C)	N concentration (mg L^−1^)	Temperature (°C)	N concentration (mg L^−1^)
**pH 3**			
25	2.86 ± 0.01	25	14.44 ± 0.72
50	25.12 ± 0.13	50	0.25 ± 0.01
70	0.43 ± 0.00	70	88.74 ± 4.44
Aqueous solvent (control)	3.72 ± 0.02	Aqueous solvent (control)	0.28 ± 0.01
**pH 7**			
25	2.57 ± 0.13	25	0.18 ± 0.01
50	16.30 ± 0.82	50	0.26 ± 0.01
70	1.21 ± 0.06	70	102.65 ± 5.13
Aqueous solvent (control)	3.72 ± 0.19	Aqueous solvent (control)	0.28 ± 0.01
**pH 11**			
25	54.49 ± 2.72	25	692.90 ± 34.65
50	376.90 ± 18.85	50	70.70 ± 3.54
70	395 ± 0.20	70	294.40 ± 14.72
Aqueous solvent (control)	3.72 ± 0.19	Aqueous solvent (control)	0.28 ± 0.01

Since minor nitrogen contamination can be derived from aqueous solvent, a control sample of aqueous solvent media only was also checked.

### Active-site density

The ability of fluorescein to bind specifically to quaternary ammonium salts was utilized to determine the active-site density of the C18-TSA-modified nonwovens. The materials were immersed in a 1% aqueous fluorescein solution and fluorescein sodium salt was coordinatively bound to the TSA moieties. After 24 h, the samples were washed with water and as expected, the control pristine PE/PP remained uncolored, whereas PE/PP-TSA and PE/PP-F-TSA turned yellow. The next step involved displacement of the TSA-coordinated fluorescein by the competitive quaternary ammonium salt cetyltrimethylammonium chloride. The concentration of fluorescein released to the solution was estimated by UV–Vis measurements.

**Figure 5:**
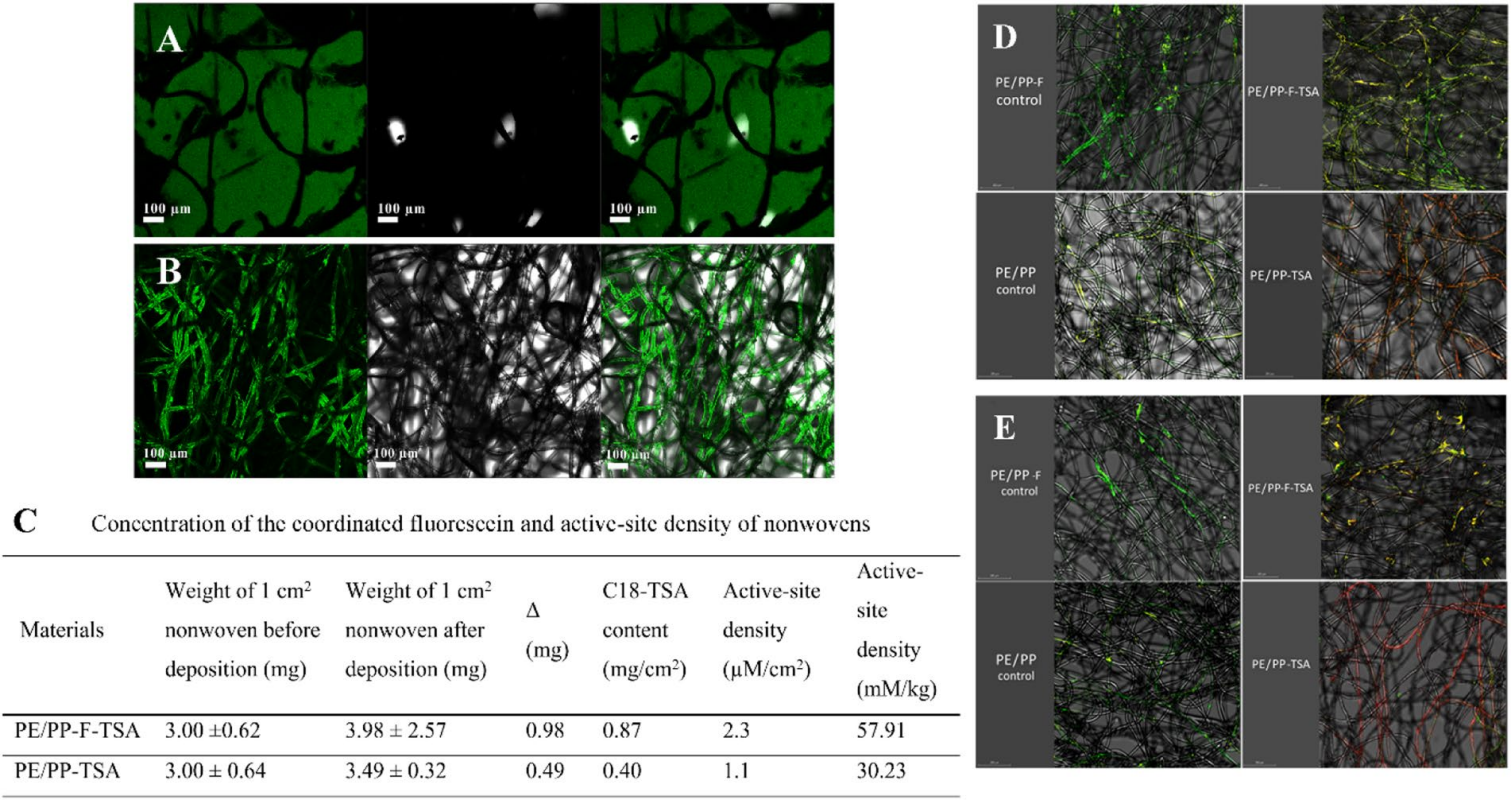
Confocal microscopy images of PE/PP-F-TSA (**A**) and PE/PP-TSA (**B**). Left images show the fluorescein-colored fibers and center images are dark-field; right images show their superposition; Concentration of the coordinated fluorescein and active-site density of nonwovens (**C**); Live/Dead staining of *Salmonella* (**A**) and *Bacillus* (**B**) on control and TSA-modified nonwovens after 24 h incubation. Live bacteria are stained green, and dead bacteria are stained red. Live and dead bacteria in close proximity produce a yellow color (**D** and **E**).

The PE/PP-F-TSA nonwovens demonstrated a higher fluorescein concentration (2.3 µM cm^−2^) than the air-ozonolysis-activated PE/PP-TSA (1.1 µM cm^−2^), which could be explained by the adsorption of fluorescein to the functional groups of the commercial finishing (Fig. 5C). Confocal microscopy measurements of PE/PP-TSA and PE/PP-F-TSA, performed after coordination of fluorescein but prior to its displacement with cetyltrimethylammonium chloride, revealed that in PE/PP-TSA, fluorescein is bound directly to the material's fibers, whereas in PE/PP-F-TSA, the binding mode is less well-defined (Fig. 5A,B).

### Antimicrobial activity

Confocal microscopy studies with Live/Dead staining of *S. enterica* (Gram negative) and *B. subtilis* (Gram-positive) cells revealed that after contact with PP-PE-TSA, most of the bacteria were dead and stained red. The PE/PP-TSA nonwovens prepared by air-ozonolysis activation followed by covalent linkage of C18-TSA seems more effective at killing the bacteria than PE/PP-F-TSA nonwovens, prepared using active finishing (Fig. 5D,E), however, further quantitative studies are needed to compare their effect.

## Discussion

In this work, two feasible pathways for modification of the polyolefins PE and PP to form active nonwoven materials were presented. In the first method, the safe O_2_-free air-ozonolysis method was used for polyolefin surface activation, followed by covalent linkage of the bioactive agent, resulting in contact-active nonwovens that also demonstrated excellent stability. In the second approach, the polyolefin surface was not activated; instead, the bioactive agent was directly attached to a commonly used commercial finishing. To the best of our knowledge, this is the first time that a routine finishing treatment has been used to form contact-active nonwovens.

The materials prepared by two approaches were comprehensively characterized. ATR-FTIR measurements unambiguously confirmed successful C18-TSA grafting (Fig. 2). The spectrum of pristine PP/PE nonwoven had two distinct aliphatic peaks at 2916 and 1470 cm^−1^, corresponding to C–H stretching and C–H bending, respectively ^24^. In the first method, air-ozonolysis activation resulted in a new carboxylic peak at 1717 cm^−1^. Upon C18-TSA conjugation, a new peak associated with Si–O–C bond appeared at 1060 cm^−1^. In the second approach, the new peaks associated with amide bond formation that correspond to N–H stretching at 3380 cm^−1^ and right-shifted carboxylic stretch at 1647 cm^−1^ ^28^ can be seen in the spectrum of PE/PP-F-TSA. The presence of carboxylic peak in PE/PP-TSA, which was modified using the first air-ozonolysis approach, reveals that some carboxylic groups did not react. (Trimethoxysilyl)propyl-octadecyl-dimethyl-ammonium chloride (C18-TSA) binding does not take place on each functional group because of a steric hindrance. The presence of carboxylic peak in PE/PP-F-TSA, which was modified using the second active finishing-based approach, is due to the unreacted functional groups of the finishing. These functional groups allowed C18-TSA linkage, but also here C18-TSA binding does not take place on each functional group because of a steric hindrance. In addition to the ATR-FTIR analysis, thermogravimetric studies, differential scanning calorimetry and N-atom analysis corroborated C18-TSA grafting, while the attached quaternary ammonium units were quantified by active site density studies.

Changes in water contact angle measurements were obtained upon the modification processes. The ozonolysis oxidation led to a formation of polar carboxylic groups which are responsible for the decreasing of the water contact angle in PE/PP_ox_ nonwovens. On the other hand, the reduction treatment increased the hydrophobicity of nonwovens due to the formation of the less polar hydroxyl groups. The conjugation of C18-TSA resulted in 120.8° and 111.5° for PE/PP-TSA and PE/PP-F-TSA, respectively. The C18-TSA conjugation decreased the surface energy due to the hydrophobic nature of the C18 backbone in both in the ozonolysis activated and in the non-activated nonwovens. The modified polymers' hydrophobic properties, can are also verified by thermogravimetric analysis that shows very low water absorbance, up to 1.6%.

Both methods produce antimicrobial nonwovens, as observed by the Live/Dead staining technique; however, further research is required to evaluate the antibacterial activity of the two fabrics against a range of bacterial pathogens using quantitative analysis. The stability study reviled that the covalent linkage of quaternary ammonium salt can provide a stronger binding comparing to the salt grafting through the commercial finishing.

Thus, the first method led to highly safe and stable nonwovens and could be relevant for biomedical, hygiene, and cosmetic applications. In the second method, the use of commercial finishing significantly simplified the modification process, enabling the production of low-cost active nonwovens that might be used, for instance, for sanitizing wipes. In conclusion, both methods can be used to synthesize nonwovens with the desired properties, and the end user can choose the most suitable method, depending on the product's use.

## Methods

### Materials

Bicomponent PE/PP nonwovens were kindly donated by Shalag Company (Israel). The following analytical-grade chemicals were purchased from Sigma Aldrich (St. Louis, MO) and were used without further purification: sodium borohydride (NaBH_4_; ≥ 98%), lithium aluminum hydride (LiAlH_4_; 95%), ethanol (absolute, 95%), tetrahydrofuran (THF) (HPLC-grade), (trimethoxysilyl) propyl-octadecyl-dimethyl-ammonium chloride (C18-TSA; 72%), cetyltrimethylammonium chloride (98%) and fluorescein sodium salt (≥ 98%). The commercial finishing agent PHP10 was produced by Schill + Seilacher GmbH (Germany) and was kindly donated by Avgol Ltd. (Israel).

### Synthesis

#### Ozonolysis of PE/PP to form oxidized films (PE/PP_ox_)

Ozonolysis was performed in an ozonator (International Application no. PCT/IL96/00090, International Publication no. WO 97/0907/1) invented by Uri M. Peiper et al. at the Institute for Agricultural Engineering (Agricultural Research Organization, Israel) and further developed by Sterilion Ltd. (Ramat Hasharon, Israel). PE/PP films (0.87 g) were treated in the ozonator for 24 h at room temperature (RT). The PE/PP_ox_ was washed with distilled water three times and dried in a desiccator under vacuum (22 mm Hg, 25 °C) overnight.

Reduction of PE/PP_ox_ to form reduced films (PE/PP_red_)PE/PP_ox_ (0.87 g) was added to a stirred solution of LiAlH_4_ (0.88 g, 5.27 mmol in 200 mL of dry THF. The reaction was performed at room-temperature (RT) overnight with constant magnetic stirring. The film was removed from the solution and washed with THF and ethanol to remove unreacted salt. The obtained film was dried in a desiccator under vacuum (22 mm Hg, 25 °C) overnight.

#### Reaction with quaternary ammonium salts to form TSA-conjugated PE/PP films (PE/PP-TSA)

The PE/PP_red_ films were dipped in a water mixture containing C18-TSA (3.975 mL of 71.29 mmol C18-TSA and 51.025 mL water) at RT for 24 h. Upon withdrawal from the quaternary ammonium salt solution, the samples were washed three times for 10 min each in water, using a Bransonic cleaner bath sonicator, and dried in a desiccator under vacuum (22 mm Hg, 25 °C) overnight.

#### Synthesis of contact-active nonwovens through the formation of active finishing (PE/PP-F-TSA)

Trimethoxysilylpropyl octadecyldimethyl ammonium chloride (3.975 mL 71.29 mmol), 41.025 mL water, and 10 mL of 5% aqueous solution of PHP10 were added to 0.9 g PE/PP. Acetic acid was added to adjust the pH to 4.0. The reaction mixture was stirred for 24 h at RT. The resulting film (PE/PP-F-TSA) was vigorously washed with water and dried in a desiccator under vacuum (22 mm Hg, 25 °C) overnight.

### Characterization

#### Attenuated total reflectance–Fourier transform infrared (ATR-FTIR) spectroscopy

ATR-FTIR spectra of the pure and modified nonwovens films were obtained using a FTIR spectrometer (Nicolet IS5) in transmittance mode.

#### Contact angle

Water-droplet contact angles on the sample surfaces were measured by a KRÜSS DSA 100 model drop shape analyzer. The image of a water droplet placed on the sample surface was captured with a CCD camera and then analyzed using DSA3 software. The contact angles of the right and left sides of the drop, and the average contact angle, were determined. The measurements were performed at RT by injecting 4 μL distilled water onto the surface of the film, and three drops per surface were evaluated.

#### Morphological study

The morphology of the nonwovens was investigated by high-resolution scanning electron microscopy (SEM). The SEM micrographs were recorded using a MIRA3 scanning electron microscope (TESCAN, Brno, Czechia). The nonwoven samples were placed on the stub and, to avoid charging problems, fibers were coated with Pd/Au for 30 s in an argon environment using a Q150T ES coater with turbomolecular pump (Quorum Technologies, Lewes, UK).

#### Thermogravimetric analysis (TGA)

Degradation of the materials was observed as a linear increase in temperature with a PerkinElmer (Waltham, MA) TGA 8000 thermogravimetric analyzer. The initial mass varied between 2 and 5 mg, the temperature range was 30–750 °C, and the heating rate was 10 °C min^−1^.

#### Differential scanning calorimetry (DSC)

Phase-transition thermograms were recorded with a PerkinElmer DSC6000 differential calorimeter. Sample mass varied between 5 and 10 mg. The samples were sealed in aluminum pans. Studies were performed between 0 and 200 °C with heating at 10 °C min^−1^.

#### Determination of active-site density

Active-site density was determined using a previously described fluorescein method^29^. Briefly, the activated PE/PP-TSA and PE/PP-F-TSA films were incubated in 2 mL of a 1% (w/v) solution of fluorescein sodium salt in water for 24 h at 30 °C. Pristine PE/PP films served as controls and were also treated with fluorescein to verify that there is no background absorption of the dye. Then the films were washed with water (3 × 10 mL) using a Bransonic cleaner bath sonicator (10 min each wash). The PE/PP films remained uncolored, while the PE/PP-TSA and PE/PP-F-TSA films turned yellow. The films were placed in 2 mL of 2.5% (w/v) cetyltrimethylammonium chloride (98%) in distilled water and vigorously shaken for 24 h at 50 °C to desorb the dye. Absorption of the coordinated fluorescein, which was decomplexed and dissolved by this treatment, was measured using a spectrophotometer (Jenway 6505 UV–Vis spectrophotometer, Shimadzu, Tokyo, Japan). Fluorescein was excited with a 470 ± 20 nm excitation filter and a 530 ± 20 nm bandpass emission filter. The absorbance of the resultant solution was measured at 499 nm. Fluorescein concentration was calculated from the Beer–Lambert equation and used to quantify the active-site density of the modified materials. An independently determined extinction coefficient of fluorescein was obtained using a calibration curve (R^2^ = 0.991) and was established to be 69.16 mM^−1^ cm^−1^, which correlated with the previously reported value^30^. All microscope measurements were performed using a confocal laser-scanning microscope (Olympus IX81, Tokyo, Japan) with a 10X objective and a numerical aperture of 0.4.

To determine the activesite site density per weight of the fabric, samples of the 1 cm × 1 cm PE/PP, PE/PP-F, PE/PP-TSA and PE-PP-F-TSA films were subjected to the same fluorescein treatment. After the fluorescein desorption, the absorption of the coordinated fluorescein was measured by UV–Vis spectrophotometry and the fluorescein concentration was calculated from the Beer–Lambert equation and used to quantify the active-site density as in the previous case. The mass of all samples was recorded before and after the treatment, and the mass of C18-TSA loaded on the TSA-conjugated nonwovens was calculated by subtracting the weights of the TSA-conjugated and unconjugated samples. The active site density was calculated as molar ratio per square centimeter and/or kilogram of fabric.

#### Stability studies

To determine the stability of TSA-conjugated nonwovens, the quantity of released nitrogen was measured using a total organic carbon analyzer (TOC-L, Shimadzu). This quantity was used to estimate the concentration of TSA released from the nonwovens. The measurements were performed in an aqueous solution under various conditions. Samples of 2 cm × 2 cm were immersed for 72 h in 40 mL distilled water or buffers with pH ranging from 3 to 11. Investigations were performed at temperatures between 25 and 70 °C. Following the incubation, the samples were squeezed, and the solution was analyzed for the presence of nitrogen. In all measurements, distilled water served as the control.

#### Water-passage test

As a measure of the treated nonwovens' hydrophilicity, the water-passage time of a test liquid was determined. The tested sample was placed on top of a beaker and firmly held at the edges to ensure nonwoven stretch. Then, 5 mL of distilled water was transferred through the nonwoven. The time required for liquid permeation through the nonwoven fabric was measured with an accuracy of 0.01 s and the results are mean values of triplicate experiments.

#### Antimicrobial-activity studies

To test the killing ability of the surfaces, two bacteria—the Gram-positive *Bacillus subtilis* PY79 (donated by Dr. M. Shemesh, Agricultural Research Organization, Volcani Center), and the Gram-negative *Salmonella enterica* sv. *typhimurium* (SL1344) were used. Bacteria were maintained as glycerol stocks and stored at 80 °C. For each experiment, a fresh culture of bacteria was used: the bacteria were grown in Luria–Bertani (LB; 10 g Bacto-peptone, 5 g yeast extract, 10 g NaCl; Difco Laboratories, Sparks, MD) for 18–20 h at 37 °C with shaking (150 rpm) to the stationary phase. For the experiments, the bacteria were washed twice with sterile distilled water by centrifugation at 2,700* g* for 10 min, and the pellet was resuspended in sterile distilled water.

Samples (1 cm × 1 cm) were contaminated by spot inoculation on the surface with 10 µL of ~ 8 log10 colony-forming units of each bacterium. The nonwovens were air-dried for 1 h at RT, and stored at the same temperature for 24 h in 50-mm Petri dishes. Bacterial viability on the nonwovens’ surfaces was tested following 24 h incubation at RT, using the LIVE/DEAD BacLight Bacterial Viability Kit (Invitrogen, Carlsbad, CA), as instructed by the manufacturer. Confocal laser-scanning microscopy (Olympus IX81), using a 10X objective with a numerical aperture of 0.4, was performed to visualize the stained *Salmonella* and *Bacillus* bacteria. Dead bacterial cells were visualized using an excitation wavelength of 543 nm and a BA560–600 nm emission filter, and live bacteria were visualized at 488 nm excitation wavelength with a BA505–525 nm emission filter. Transmitted-light images were obtained using Nomarski differential interference contrast.
